# A prediction rule for shoulder pain related sick leave: a prospective cohort study

**DOI:** 10.1186/1471-2474-7-97

**Published:** 2006-12-06

**Authors:** Ton Kuijpers, Daniëlle AWM van der Windt, Geert JMG van der Heijden, Jos WR Twisk, Yvonne Vergouwe, Lex M Bouter

**Affiliations:** 1 Institute for Research in Extramural Medicine, VU University Medical Center, Amsterdam, The Netherlands; 2 Department of Allied Health Care Research, Amsterdam School of Allied Health Care Education, The Netherlands; 3 Care and Public Health Research Institute, Maastricht University, The Netherlands; 4 Primary Care Musculoskeletal Research Centre, Keele University, Keele Staffordshire, UK; 5 Julius Center for Health Sciences and Primary Care, University Medical Center, Utrecht, The Netherlands; 6 Department of Clinical Epidemiology and Biostatistics, VU University Medical Center, Amsterdam, The Netherlands

## Abstract

**Background:**

Shoulder pain is common in primary care, and has an unfavourable outcome in many patients. Information about predictors of shoulder pain related sick leave in workers is scarce and inconsistent. The objective was to develop a clinical prediction rule for calculating the risk of shoulder pain related sick leave for individual workers, during the 6 months following first consultation in general practice.

**Methods:**

A prospective cohort study with 6 months follow-up was conducted among 350 workers with a new episode of shoulder pain. Potential predictors included the results of a physical examination, sociodemographic variables, disease characteristics (duration of symptoms, sick leave in the 2 months prior to consultation, pain intensity, disability, comorbidity), physical activity, physical work load, psychological factors, and the psychosocial work environment. The main outcome measure was sick leave during 6 months following first consultation in general practice.

**Results:**

Response rate to the follow-up questionnaire at 6 months was 85%. During the 6 months after first consultation 30% (89/298) of the workers reported sick leave. 16% (47) reported 10 days sick leave or more. Sick leave during this period was predicted in a multivariable model by a longer duration of sick leave prior to consultation, more shoulder pain, a perceived cause of strain or overuse during regular activities, and co-existing psychological complaints. The discriminative ability of the prediction model was satisfactory with an area under the curve of 0.70 (95% CI 0.64–0.76).

**Conclusion:**

Although 30% of all workers with shoulder pain reported sick leave during follow-up, the duration of sick leave was limited to a few days in most workers. We developed a prediction rule and a score chart that can be used by general practitioners and occupational health care providers to calculate the absolute risk of sick leave in individual workers with shoulder pain, which may help to identify workers who need additional attention. The performance and applicability of our model needs to be tested in other working populations with shoulder pain to enable valid and reliable use of the score chart in everyday practice.

## Background

Shoulder pain is common with a one-year prevalence ranging between 5% and 47% [1,2]. [3,4] In a general practice population a one-week prevalence of 21% and one-month prevalence of 18% were reported [5]. A Finnish study [6] reported a one-year incidence of shoulder pain of 14% among forestry workers. Shoulder pain has an unfavourable outcome in many patients. About 50% of all new episodes of shoulder disorders presented in primary care show complete recovery within 6 months[7-9], while after one year this proportion increases to only 60%[8]. In an occupational study a one year persistence of shoulder pain of 55% was reported[6]. Especially in a working population loss of productivity and sick leave may be an important consequence of shoulder pain[10]. In a systematic review of the literature we summarized the available evidence from 16 studies focusing on the prognosis of shoulder pain [11]. Only six studies were of relatively high quality. In an occupational setting strong evidence for predicting poorer outcome was only found for age (45–54 years). There were no studies of sufficient quality, which studied sick leave as unfavourable outcome [11]. Evidence for the prognostic value of psychological factors or the psychosocial work environment was lacking [2].

It has been suggested that many factors play a role in the occurrence of new episodes of shoulder pain, including work-related physical and the psychosocial factors [12,13]. Individual factors seem to determine whether persons with these musculoskeletal complaints take sick leave [13]. Possibly, these 0k leave, and may help to identify workers who need additional attention. We performed a cohort study among workers who had presented shoulder pain to their general practitioner, and followed them for 6 months. Our objective was to develop a score chart to identify workers who will report at least 1 day of shoulder pain related sick leave during 6 months following first consultation for their complaints.

## Methods

### Study population

Between January 2001 and June 2003, 103 general practitioners recruited workers with a new episode of shoulder pain in three geographic areas in the Netherlands (Amsterdam, Groningen and Maastricht). Workers were selected if they were 18 years or older of age, had a paid job (all kind of workers, either on a permanent or a temporary contract), and had not consulted their GP or receive any form of treatment in the preceding 3 months for the afflicted shoulder. Sufficient knowledge of the Dutch language was required to complete written questionnaires. Exclusion criteria were serious physical or psychiatric conditions (i.e. fractures or dislocation in the shoulder region; rheumatic disease; neoplasm; neurological or vascular disorders; dementia). There was no restriction with respect to type of work or occupation, or whether or not the patient was on sick leave at the time of consultation.

### Management of shoulder pain

All workers received standardised treatment according to the 1999 version of the Dutch guidelines for shoulder complaints issued by the Dutch College of General Practitioners[14,15] which consists of information on the prognosis of shoulder pain, advice regarding provoking activities, and stepwise treatment consisting of paracetamol, NSAIDs, corticosteroid injection or referral for physiotherapy. The GP made the decision regarding the content of treatment based on duration and severity of pain and disability. The participating general practitioners were educated and trained to apply treatment according to this guideline.

Some GPs may have advised participants regarding return to work, but in the Netherlands this is primarily the task of an occupational physician.

### Prognostic factors

Within a few days after consultation all workers received a questionnaire by post and completed it (approximately 40 minutes). The questionnaire contained questions on sociodemographic variables, disease characteristics; physical activity, physical work load, psychosocial work environment, and psychological factors. Disease characteristics included pain intensity (0–10 point rating scale), shoulder pain related disability, pain onset, duration of symptoms, previous episodes of shoulder pain (i.e. having had at least one week of shoulder pain in the past), sick leave (due to shoulder pain) in the two months prior to consultation, and comorbidity. Physical activity was measured with a single question (less/equally/more active than others). A physical examination was carried out by trained assistants. Research assistants were trained thoroughly, and consistency was checked during several meetings. In some centres the research assistant was either a physiotherapist or another assistant with experience with examining patients with musculoskeletal pain.

Physical work load was measured with a self-constructed scale of 7 questions (yes/no) concerning pushing and pulling, lifting weights; working with vibrating tools, lifting weight on one shoulder, working with hands above shoulder level, repetitive movements, and sitting in the same position for a long period of time. Each item was scored positive if the participant performed the activity on at least two days a week. Factor analysis showed that the first five items reflected one dimension (total score 0–5, Crohnbach's α = 0.74). Repetitive movements and sitting were analysed as separate items.

The pychosocial work environment was assessed with five dimensions of the Job Content Questionnaire (JCQ)[1], which measures all dimensions of the widely used Demand-Control-Support model. On a four point scale (totally disagree, disagree, agree, totally agree) workers rated several aspects of their work. The JCQ consists of the dimensions quantitative job demands (5 questions, sumscore 5–20); skill discretion (5 questions, sumscore 5–20); decision authority (3 questions, sumscore 3–12); co-worker support (4 questions, sumscore 4–16) and supervisor support (4 questions, sumscore 4–16), as proposed by Karasek et al.[16,17] and clinimetrically evaluated by De Jonge et al.[18].

Previous research had shown that psychosocial factors may be important in the transition from acute to more chronic pain problems. One of the objectives of our cohort study was to test this hypothesis. The bio psychosocial model, and in particular the fear-avoidance beliefs model was used as a theoretical framework when selecting our prognostic factors. All mentioned factors are elements of these theoretical models. The following psychological variables were measured: pain coping, anxiety, depression, somatization, distress, fear-avoidance beliefs, kinesiophobia, and beliefs regarding the causes of shoulder pain. Pain coping was assessed with the 43-item Pain Coping and Cognition List (PCCL)[19], consisting of the subdomains catastrophizing (1–6 points), coping with pain (1–6 points), internal (1–6 points) and external locus of control (1–6 points). Anxiety (0–24 points), depression (0–12 points), somatization (0–32 points), and distress (0–32 points), were measured with the 50-item Four-Dimensional Symptom Questionnaire (4DSQ) [20]. Fear-avoidance beliefs were assessed using the 4-item physical activity subscale of the Fear Avoidance Beliefs Questionnaire (FABQ; 0–24) [21]. Kinesiophobia was measured using two items of the Tampa Scale for Kinesiophobia (TSK; 0–12) [22]. Participants were also asked about their beliefs regarding the possible cause of shoulder pain: unexpected movement; strain during unusal activities; overuse or strain or during regular activities; trauma; sports injury; or unclear (yes/no). Finally, our baseline questionnaire included a general one-item question regarding the presence of psychological problems: "Do you have any psychological complaints, such as distress, depression, or anxiety?" (yes/no).

Function of the shoulder joint and cervicothoracic spine were tested during a physical examination. For the glenohumeral joint active and passive abduction, passive exorotation [23], and shoulder impingement [24] were tested. Two alternative functional tests, HIB (Hand-in-back) and HIN (Hand-in-neck) [25,26] measured on a 7-point scale (score 0 = very poor range of motion, score 7 = full range of motion) were performed as well. The assistant made an estimation of the range of motion in degrees (°).

During all mobility tests self-reported pain was assessed on a 4-point scale (0 = no pain; 3 = severe pain). A factor analysis on the results of a physical examination in a similar population of patients with shoulder pain resulted in four factors: shoulder mobility, shoulder pain, neck mobility, and neck pain [27].

The factor 'shoulder mobility' consisted of 6 mobility tests: HIB, HIN, active abduction, passive abduction, external rotation, and Impingement. For calculation of the sum score (0–18 points) variables were recoded into a 4-point scale, with 0 reflecting full range of motion and 3 points reflecting very poor range of motion. HIB/HIN scores were recoded as: score 7 = 0; score 5 and 6 = 1; score 3 and 4 = 2; score 1 and 2 = 3. Abduction (active and passive) was recoded as 170–180° = 0; 140–170° = 1; 90–140° = 2; 0–90° = 3. External rotation was recoded as >80° = 0; 70–80° = 1; 50–70° = 2; <50° = 3. During the impingement test pain was measured (0 = no pain; 3 = severe pain). The factor 'shoulder pain' (0–18 points) consisted of the sum of the pain scores during the mobility tests.

The factor 'neck mobility' (0–4 points) consisted of rotation of the cervicothoracic spine in neutral, flexed, and extended position and lateral bending. These range of motion tests were scored as (1 = decreased range of motion, and 0 = no decreased range of motion). The factor 'neck pain' (0–18 points) consisted of the sum of the pain scores during flexion and extension of the neck, rotation in a neutral, flexed and extended position, and lateral bending.

### Outcome measurements

The outcome was measured by postal questionnaires at 6 weeks, 3 and 6 months. Our primary outcome measure was sick leave due to shoulder pain (yes = ≥ 1 day, no = 0 days). Secondary outcome measures were patient perceived recovery, shoulder disability, measured with the 16-item shoulder disability questionnaire (SDQ; 0–100) [28], shoulder pain (0–10 numeric rating scale) [29], and severity of the main complaint (0–10 numeric rating scale) [30]. We studied the relationship between our primary and secondary outcome measures to determine if workers reporting sick leave during follow-up showed higher levels of pain and disability.

### Analysis

Missing values of patient characteristics were imputed (approximately 2% of all required values). Imputation was based on the correlation between the variable with missing values with the other patient characteristics. Univariable logistic regression analyses were performed for all potential prognostic indicators with our primary outcome measure, i.e. sick leave during 6 months following first consultation. Variables that had a statistically significant association with the outcome (p-value ≤ 0.20) were selected for the backward selection in the multivariable analysis and checked for co-linearity. If the correlation between two potential predictors was larger than 0.5, we included in our multivariable analysis the predictor that was considered to be most relevant to the general practitioner and was easy to measure. We adopted a hierarchically approach in the variable selection in which easily obtainable predictors were included first. Therefore, variables were selected in blocks of increasing effort to obtain during consultation: 1) socio-demographic factors and disease characteristics; 2) physical factors; 3) psychosocial work environment; 4) psychological factors; 5) physical examination. Variables with the lowest predictive value were deleted from the model until further elimination of a variable resulted in a statistically significant lower model fit estimated with the likelihood ratio test (p ≤ 0.20).

Prediction models usually provide too extreme estimates when no correction is applied in the development phase. Therefore, we used bootstrap samples to estimate a 'shrinkage factor' (between 0 and 1) [31]. The regression coefficients were subsequently multiplied with this shrinkage factor to prevent the model for overfitting and overoptimism. Bootstrap samples were drawn with replacement (100 replications) from the full data set. The backward selection of variables and model fitting was repeated within each bootstrap sample. Bootstrapping techniques were also used to study the internal validity of the final prediction model [31,32]. The model's performance obtained after bootstrapping can be considered as the performance that can be expected in similar future patients. All analyses were performed using S-plus 6.1 (Insightful Corp., Seattle, WA, USA).

### Evaluation of the model

The reliability of the multivariable model was determined with the Hosmer-Lemeshow goodness-of-fit statistic [33]. Calibration of the model predictions, which is related to reliability, was assessed by plotting the predicted individual probability against the observed sick leave. For this, workers were grouped into quintiles according to their predicted probability for sick leave according to the model. The prevalence of the endpoint within each quintile represents the observed probability. The area under the receiver-operating characteristic curve (ROC) was used to assess the performance of the model in terms of accuracy of correct prediction. The ROC-curve is a plot of the true positive rate (sensitivity) against the false-positive rate (1-specificity) of the model. The curve illustrates the ability of the model to discriminate between workers with and without sick leave at subsequent cut-off points along the range of the predicted probabilities. An area under the curve (AUC) of 0.5 indicates no discrimination above chance, whereas an AUC of 1.0 indicates perfect discrimination.

### Prediction of an individual patient's risk

We developed a clinical prediction rule for sick leave during 6 months following first consultation, to provide general practitioners and occupational health care providers with an estimate of the absolute risk of sick leave for individual workers. Since we used logistic regression, the probability (P) of sick leave was predicted with P = 1/[1+ exp- (a_0 _+ b_1_x_1 _+ ...... + b_j_x_j_)]. The status of a patient for any dummy or binary variable included in the prediction rule can be either 0 or 1, while for a (semi) continuous variable it takes the actual observed value.

### Score chart

To facilitate the calculation of an individual worker's risk, we developed a score chart. We multiplied the regression coefficients by 4 and rounded them to the nearest integer to form the scores for each of the predictors. The scores of predictors which are reviewed positively are added to calculate the 'Total score'. This total score corresponds to risk of sick leave during follow-up.

The study was approved by the Medical Ethics Committee of the VU University Medical Center, Amsterdam, the Netherlands.

## Results

### Study population and follow-up

A total of 350 workers with shoulder pain in primary care completed the baseline assessment. Table 1 lists the baseline characteristics. At 6 months 298 (85%) workers returned the postal questionnaire. The drop-outs at 6 months (n = 52) showed significantly (p < 0.10) more pain of the shoulder (2.4 versus 2.3 points) and the neck (2.8 versus 2.6 points) at physical examination and less decision authority (4.4 versus 5.5 points) at baseline.

During the 6 months following first consultation 30% (89/298) of the workers reported at least one day of sick leave (primary outcome measure) because of their shoulder pain, for 25 sick leave was limited to the first 6 weeks of follow-up. 16% (47/298) of the workers reported more than 10 days of shoulder pain related sick leave in 6 months.

### Management of shoulder disorders

At first consultation most workers (n = 253, 73%) received a wait and see policy, paracetamol, or NSAIDs. Furthermore at first consultation, 35 workers (10%) received an injection with corticosteroid, 41 workers (12%) were referred for physiotherapy and 17 workers (5%) received other therapies.

### Prognostic model

The univariable associations of the determinants with shoulder pain related sick leave during the 6 months following first consultation are presented in Table 1. Only variables which showed an univariable association (p ≤ 0.20) were selected for the backward stepwise selection. Table 2 presents the variables for the prediction model after backward stepwise analysis. In the backward analysis the blocks physical factors, psychosocial work environment, psychological factors, and physical examination did not result in a better model fit. A longer duration of sick leave prior to consultation, higher shoulder pain intensity, strain (overuse) as a result of usual activities and co-existing psychological complaints were associated with a higher risk of sick leave during 6 months. The multivariable regression coefficients were additionally shrunk (shrinkage factor = 0.72) to obtain optimism corrected predictions for new workers.

### Evaluation of the model

**Figure 1:**
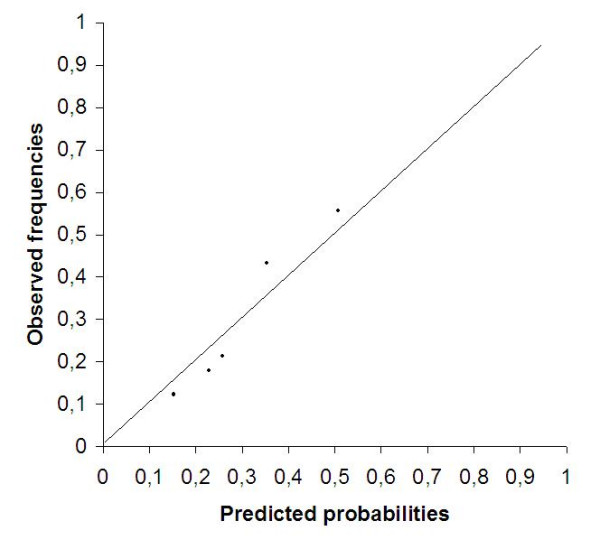
Calibration plot showing the observed probability versus the predicted probability for sick leave in workers with shoulder pain during 6 months following first consultation.

**Figure 2:**
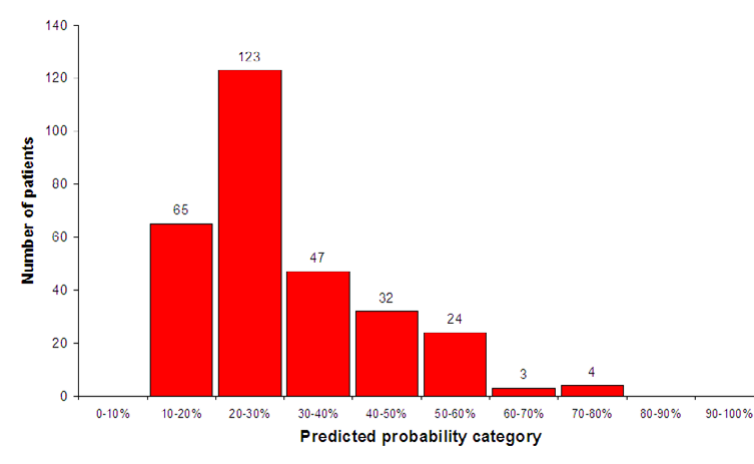
Distribution of predicted risk for sick leave during 6 months after first consultation (n = 298).

The reliability of the model was adequate, according to the Hosmer-Lemeshow statistic, with a non-statistically significant p-value of 0.13. Figure 1 shows the calibration of the prediction model. The plotted points are rather close to the 45° line, demonstrating good calibration over the whole range of the predictions. The distribution of the predicted risk ranges between 15 and 75% (Figure 2). The AUC of 0.70 (95% CI 0.64–0.76) represents satisfactory discrimination.

### Score charts

**Figure 3:**
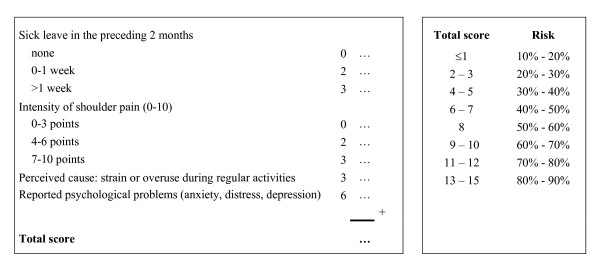
The predicted probability of sick leave during 6 months was determined by P = 1/[1+ exp - (-1.72 + 0.53 × sick leave 0–1 week + 0.77 × sick leave >1 week + 0.50 × shoulder pain (4–6 points) + 0.65 × shoulder pain (7–10 points) + 0.68 × overuse due to usual activities + 1.38 × concomitant psychological problems)]. **Instruction** If a predictor is scored positively, the given weight needs to be filled in. Subsequently the scores are added to calculate the 'Total score'. Using the table next to the score chart the risk (%) of sick leave for an individual patient can be determined based on his/her total score.

Figure 3 shows the score chart for predicting sick leave during 6 months after first consultation. As an example, a worker with sick leave at baseline of 2 weeks (3 points), a score of 5 points on the shoulder pain scale (2 points), and whose shoulder pain is caused by usual activities (3 points), has a total score of 8 points corresponding to a risk of 50–60% for sick leave during 6 months after consultation.

## Discussions and Conclusions

In this study we developed a score chart to predict shoulder pain related sick leave during 6 months following first consultation. A longer duration of sick leave prior to consultation, shoulder pain, a perceived cause of strain or overuse during regular activities and co-existing psychological complaints were associated with a higher risk of sick leave during 6 months following consultation.

### Outcome

Even though 30% of all participants reported sick leave due to shoulder pain in the 6 months following consultation, only 16% reported sick leave during at least 10 days. This seems to indicate that in our population, despite persisting pain and disability in many workers, sick leave was neither a very frequent nor long-lasting problem. An explanation could be that we did not select a specific occupational group with a higher risk of sick leave due to shoulder pain. Our population contained all kind of workers, irrespective of physical work load or the psychosocial work environment. Persistent symptoms, pain, and disability were also found among those who remained at work (data not shown). It may be possible that shoulder pain, sleeping problems, disability did cause loss of productivity in workers who continued working. However, were unable to assess measures of performance or productivity in this study.

### Prognostic factors

The conceptual model we used for selecting candidate predictors during the design of our study was a biopsychosocial model of pain, in which factors potentially influencing the perception of pain are described: clinical disease characteristics, psychological factors (pain cognitions, fear avoidance beliefs, distress), physical work load, and the psychosocial work environment. Evidence for the influence of these factors on the prognosis of shoulder pain is, as yet, limited. In a systematic review [11] of the literature we found strong evidence only for age (45 – 54 years) as a predictor for poorer outcome in occupational populations. In our study no association was found for age with sick leave. It has previously been suggested that psychological factors, such as inadequate pain cognitions and pain behaviour are likely to predict a poor outcome of painful musculoskeletal conditions [2]. Furthermore, there is evidence that the psychosocial work environment (e.g. decision authority and job satisfaction) [12] and heavy physical work load (e.g. pushing and pulling, repetitive work) [12,34] may be associated with an increased risk of new episodes of shoulder pain. Our study, however, shows that these risk factors do not predict sick leave among workers who have consulted a GP for their shoulder pain. We assessed physical work load with a simple checklist consisting of items that have been shown to predict the occurrence of shoulder pain. A more extensive or specific measurement of physical work load might have revealed stronger associations between physical exposures and shoulder pain related sick leave, but such an assessment was not feasible within this large primary care population. Participants were not recruited in specific occupational settings, meaning that a wide variety of jobs and tasks were performed.

The baseline scores on psychological and work-related psychosocial variables were generally low in our population. Significant univariable associations with sick leave during follow-up were found for several factors (distress, somatisation, catastrophising, fear avoidance, decision authority, and co-worker support), but in a multivariable model these factors had little to add to a few general and simple questions regarding the presence of psychological complaints, strain or overuse, pain intensity and sick leave at baseline. The prediction rule, consequently, contains easy to derive predictors. The prediction rule rather accurately estimates the risk of sick leave in individual workers with shoulder pain, and may help to identify workers who need additional attention.

### Model fit and discrimination

The calibration plot (Figure 1) showed that the predicted probability categories were close to the ideal line. This indicates that in general the model was rather well calibrated over the complete range of predicted probabilities. The optimism corrected AUC of 0.70 implied satisfactory discrimination between shoulder patients with and without sick leave.

We did not enrol a 'true' inception cohort, in the sense that all patients were enrolled very early in the course of their disease Our cohort was a mixed population of patients who still worked at the time of consultation, had recently reported sick leave, or already had been off work due to shoulder for a long period of time. It would be informative to develop separate prognostic models for workers without current sick leave, and those with longstanding or previous sick leave at presentation in order to assess whether the effects of potential predictors such as pain intensity or psychological problems differ in relevant subgroups. In our population, however, the number of patients reporting previous sick leave was small. Identification of relevant subgroups is an important next step in research, which may result in the development of prediction rules with optimal performances in terms of calibration and discrimination.

### Generalisability

GPs are consulted about two times each month for a new episode of shoulder pain [3]. Given exclusion criteria we might have expected about 1 patient each month per practice. Not all practices participated for 2,5 years, for example, 30 practices in one geographical were involved in the study for a period of 8 months. It is difficult to estimate how many eligible patients were not invited to participate, but it is not unlikely that our GPs enrolled only half of all eligible subjects. Few patients refused to participate. GPs indicated that it was mostly time constraints or the fact that they forgot to invite patients. The characteristics of our study population are not dissimilar to those of another population with neck or shoulder problems recruited in Dutch general practice [35]. We have therefore no reason to assume that our sample constitutes a highly selective sample of workers'

The response to the questionnaires was satisfactory in our study. Yet, selective dropout can be an important drawback even if the proportion of loss to follow-up is small. Dropouts showed higher pain scores and less decision authority, but the absolute differences at baseline between responders and drop-outs were small. Therefore, we do not believe that selection bias had a strong impact on the presented results, and we assume that the results may be generalised to other patients consulting their general practitioner for shoulder pain.

We recruited patients with shoulder pain in primary care, which resulted in a heterogeneous working population. The characteristics of our population may certainly differ from workers with shoulder pain in more specific occupational settings. This does not necessarily mean that our prediction rule is not a viable tool in other populations. However, before considering implementation of our prognostic model (i.e. score chart) in general or occupational practice, the generalisability ('external validity') of the model needs to be tested in other populations of workers with shoulder disorders [36]. First, its generalisability to other working populations can be tested. If satisfactory, the generalisability to a community sample or secondary care populations may be tested.

### Score charts

The score charts in our study were developed to provide primary or occupational care physicians with an easy tool to predict the risk of shoulder pain related sick leave. The score charts consist of easy 'yes or no' questions, and a simple question for the intensity of shoulder pain (0–10). The strength of a prediction rule is the possibility to calculate a risk at individual patient level. However, some variation may be expected when individual risks are calculated. In order to take some of this uncertainty into account, we composed risk categories with ranges of 10 percent. Such categories may be easier to communicate with patients than point estimates with 95% confidence intervals. Physicians may use the prediction rule programmed in a PC or PDA to calculate the risk of sick leave for workers with shoulder pain. Because we feel that not every physician may have direct access to such equipment, we developed a score chart to enable easy implementation in everyday practice. The applicability and usefulness of the score chart needs to be further tested in a clinical practice setting.

We built a prediction model with the purpose of risk stratification, i.e. to distinguish between working patients at high or low risk of reporting sick leave because of shoulder pain. The prediction rule may enable clinicians to identify workers with shoulder pain in at risk for sick leave. Possibly a higher risk is a sign for the health care provider to discuss work related issues.

## Competing interests

The author(s) declare that they have no competing interests.

## Authors' contributions

TK carried out the analysis and drafted the manuscript. DAW, GJH, JT, YV, LMB, and participated in the design and drafting of the study and read and approved the final manuscript.

## Pre-publication history

The pre-publication history for this paper can be accessed here:



## Figures and Tables

**Table 1:** Baseline characteristics of a working population with shoulder disorders (n = 350), and univariable associations with sick leave (yes/no) during 6 months following first consultation in general practice

**Variable**	**n (%)^@^**	**OR**	**95% CI**	**p^a^**
*Demographic*				
Age (years); mean (SD)	45 (11)	1.0	1.0, 1.0	0.26
Gender: male	193 (55)	0.9	0.5, 1.4	0.56
Education				0.02
Low*	98 (28)	-		
Middle	148 (43)	0.5	0.3, 0.9	
High	99 (29)	0.4	0.2, 0.9	
				
*Disease characteristics*				
Duration of complaints				0.77
0–6 weeks*	139 (40)	-		
7–12 weeks	77 (22)	1.1	0.6, 2.1	
>3 months	134 (38)	0.9	0.5, 1.5	
Sick leave at baseline in preceding 2 months				<0.001
0 weeks*	254 (74)	-		
≤1 weeks	44 (13)	1.8	0.9, 3.9	
>1 weeks	46 (13)	3.3	1.6, 6.9	
Gradual onset (vs. acute)	212 (61)	1.0	0.6, 1.6	0.89
Opinion regarding cause				
Unexpected movement	16 (5)	1.7	0.5, 5.6	0.36
Strain/overuse: unusual activities	56 (16)	0.5	0.2, 1.1	0.08
Strain/overuse: regular activities	99 (28)	2.4	1.4, 4.1	<0.001
Injury	15 (4)	4..4	1.4, 15.3	0.01
Sport injury	22 (6)	0.5	0.1, 1.7	0.26
Unknown	133 (38)	1.4	0.9, 2.4	0.17
Shoulder complaints in the past	199 (57)	1.4	0.8, 2.3	0.19
Neck complaints in the past	165 (48)	1.5	0.9, 2.4	0.11
Dominant side involved	210 (60)	1.5	0.9, 2.4	0.15
Co-existing psychological complaints	27 (8)	5.4	2.0, 13.9	<0.001
Additional musculoskeletal complaints				
Neck/high back	119 (34)	1.6	1.0, 2.7	0.07
Low back pain	61 (17)	1.3	0.6, 2.4	0.50
Upper extremity	96 (27)	1.4	0.8, 2.5	0.21
Lower extremity	77 (22)	0.7	0.4, 1.3	0.30
Shoulder pain (0–10); mean (SD)	4.5 (2.3)			0.01
0–3 points*		-		
4–6 points		2.4	1.3, 4.5	
7–10 points		2.3	1.2, 4.5	
Shoulder disability (SDQ) (0–100); mean (SD)	58.4 (24.0)	1.9	0.8, 4.8	0.24
				
*Physical examination*				
ROM shoulder (0–18) (median, IQR)	3 (2–5)	0.7^#^	0.3, 1.6	0.74
Pain shoulder with movement (<12, ≥ 12 points)	6.3 (4.0)	2.9^#^	1.3, 6.7	0.03
ROM neck (0–4) (median, IQR)	0 (0–1.5)	1.2^#^	0.7, 2.2	0.41
Pain neck with movement (0–18) (median, IQR)	0 (0–3)	1.1	1.0, 1.3	0.01
				
*Physical factors*				
Physical work load (0–5) (median, IQR)	1.6 (1.6)	1.3	1.1, 1.5	0.01
Repetitive movements (yes/no)	279 (80)	1.0	0.6, 1.9	0.97
Sitting in static position for long duration (yes/no)	138 (40)	1.2	0.7, 1.9	0.54
Physical activity in comparison to others				0.09
more active*	130 (37)	-		
equally active	158 (45)	1.5	0.9, 2.7	
less active	60 (17)	2.2	1.1, 4.5	
				
*Psychological factors*				
Coping; mean (SD)				
Catastrophizing (1–6)	2.2 (0.8)	1.6	1.1, 2.2	0.01
Coping with pain (1–6)	2.9 (1.0)	2.6^#^	0.4, 17.9	0.44
Internal locus of control (1–6)	3.4 (0.9)	1.5^#^	0.4, 5.5	0.76
External locus of control (1–6)	3.1 (0.9)	1.9^#^	0.3, 14.2	0.76
4DSQ (median, IQR)				
Distress (0–32)	0 (0–2)	4.4^$^	1.3, 15.5	0.01
Depression (0–12)^b^	0 (0-0)	-		
Anxiety (0–24)^b^	0 (0-0)	-		
Somatization (0–32)	2 (0–4)	3.0^$^	1.0, 9.1	0.05
Fear-avoidance (0–24); mean (SD)	14.4 (5.0)	1.1	1.0, 1.1	0.04
Kinesiophobia (0–12); mean (SD)	3.3 (3.4)	1.7^#^	0.5, 5.3	0.07
				
*Psychosocial work environment*				
Quantitative job demands (5–20); mean (SD)	12.8 (2.7)	1.4^#^	0.6, 3.0	0.68
Skill discretion (5–20); mean (SD)	15.3 (2.8)	1.8^&^	0.6, 5.3	0.57
Decision authority (3–12); mean (SD)	9.4 (1.8)	0.8	0.7, 0.9	<0.001
Co-worker support (4–16); mean (SD)	12.3 (2.0)	10.1^&^	1.1, 92.4	0.07
*Supervisor support (4–16); mean (SD)*	11.2 (2.5)	2.0^&^	0.9, 4.2	0.20

SD = standard deviation; IQR = Inter quartile range; ROM = Range of Motion; 4DSQ = Four-dimensional symptom questionnaire. ^a^Variables with a univariable p-value ≤ 0.20 were selected for the multivariable analysis. ^b^ORs haven't been computed due to empty cells in the cross-tables. ^@^or otherwise stated *Reference category. ^#^In case of non-linear associations continuous variables were divided into categories. The table presents the Odds Ratio (OR) for the highest versus lowest category. ^&^ORs were computed for lowest versus highest categories. ^$^Variable was dichotomised. (*lowest versus middle and high category*).

**Table 2:** Multivariable model with predictors of shoulder pain related sick leave during 6 months following first consultation (n = 298)

**Variable**	**OR**	**95% CI**
Sick leave at baseline (in preceding 2 months)		
0-weeks*		
≤1 week	1.7	0.8–3.6
>1 week	2.2	1.0–4.7
Shoulder pain (0–10)		
0–3 points*		
4–6 points	1.7	0.9–3.2
7–10 points	1.9	0.9–3.9
Strain, overuse: usual activities (yes/no)	1.9	1.1–3.5
Co-existing psychological complaints (yes/no)	4.0	1.5–10.8

*Reference category
